# K^+^ Channels in Membrane Potential Dynamics and Migration of Neutrophil Granulocytes

**DOI:** 10.1002/ardp.70365

**Published:** 2026-10-16

**Authors:** Tahir Ulu, Henrik Siemer, Alexa Heuer, Sandra Schimmelpfennig, Peter Dieterich, Jesús Borrego, György Panyi, Albrecht Schwab

**Affiliations:** ^1^ Institut für Physiologie II, University Münster Münster Germany; ^2^ Institut für Physiologie, TU Dresden Dresden Germany; ^3^ Department of Biophysics and Cell Biology Faculty of Medicine, University of Debrecen Debrecen Hungary

**Keywords:** chemotaxis, KCa3.1, Kir2.1, Kv1.3, LTB_4_, membrane potential dynamics, Neutrophil granulocyte

## Abstract

Neutrophil granulocytes exhibit dynamic changes in membrane potential upon stimulation with the complement component C5a. It is not well‐defined which ion channels are responsible for membrane potential dynamics in neutrophils, although ion channel signaling has been linked to neutrophil behavior. Here, we investigated the contribution of K^+^ channels that are important players in different immune cells: K_Ca_3.1, K_ir_2.1, and K_V_1.3. We studied their roles in membrane potential regulation, chemotaxis, and LTB4 secretion using live‐cell imaging, analytical methods, and mathematical modeling. We identify membrane potential dynamics as an important regulatory axis in neutrophil migration. K_ir_2.1 is a key regulator of resting membrane potential. K_ir_2.1 blockade depolarizes the membrane potential of neutrophils by ~30 mV to −41.9 mV, and it delays and attenuates the C5a‐induced depolarization. In contrast, K_Ca_3.1 and K_V_1.3 inhibition neither modifies the resting membrane potential nor the C5a‐induced depolarization. Nonetheless, the inhibition of all three K^+^ channels impairs chemotaxis toward C5a, in part by a reduced LTB_4_ secretion and/or possibly by non‐conductive properties related to the interactome of the three K^+^ channels. Together, these findings demonstrate that K_Ca_3.1, K_V_1.3 and K_ir_2.1 channels play distinct but critical roles in neutrophil function and inflammatory signalling.

## Graphical abstract

Neutrophil membrane potential dynamics regulate directed migration. K_ir_2.1 regulates neutrophil membrane potential and indirectly LTB_4_ secretion. Together with K_Ca_3.1 and K_V_1.3, it is required for efficient chemotaxis. K_Ca_3.1 and K_V_1.3 influence neutrophil migration without altering membrane potential, potentially through reduced LTB_4_ release (K_Ca_3.1) and/or through yet to be defined non‐conductive channel functions (K_Ca_3.1, K_V_1.3).

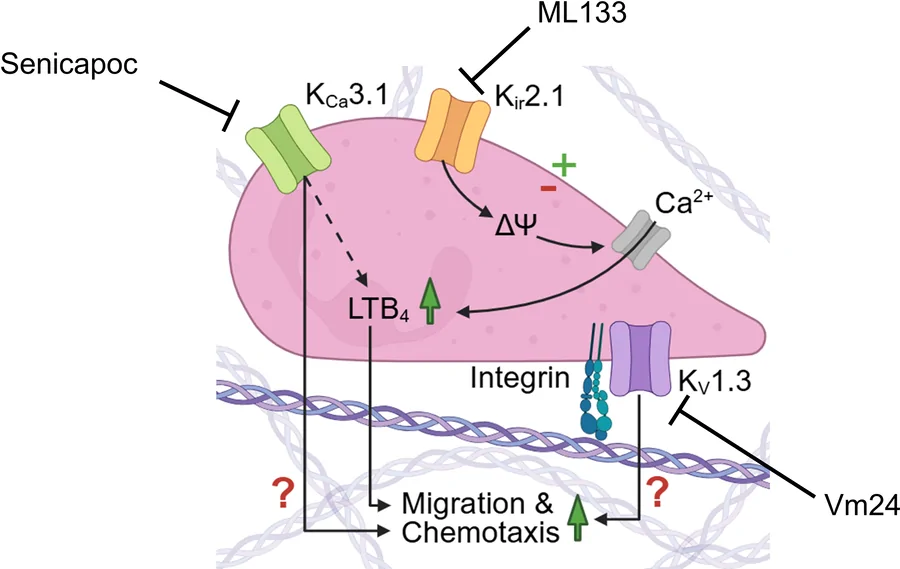

## 1 Introduction

The initial response of neutrophil granulocytes is critical for the coordinated function of the innate immune system. Their rapid recruitment and diverse effector functions including chemotaxis, phagocytosis, production of reactive oxygen species (ROS) during the respiratory burst, degranulation, and NETosis represent a central first line of defense against invading pathogens and an important driver of inflammatory responses [1]. Among these functions, chemotaxis is of particular relevance, as it enables neutrophils to efficiently accumulate at sites of infection or tissue injury.

Chemotaxis describes the directed movement of cells along gradients of chemoattractants. Important endogenous chemoattractant signals for neutrophils include, among others, complement component C5a and lipid mediators such as leukotriene B4 (LTB_4_), which orchestrate both the initiation and amplification of neutrophil recruitment [2, 3]. Mechanistically, chemotaxis relies on a tightly regulated interplay between gradient sensing, intracellular signaling pathways, and dynamic cytoskeletal remodeling, which together drive cell polarization and motility [4, 5].

In addition to “classical” signalling pathways, ionic signaling is emerging as a regulator of neutrophil behavior. The relevance of ion channel‐dependent signaling in neutrophils is illustrated by several studies, including our own, that described neutrophils with “ion channel phenotypes”. Thus, H_V_1 channels [6, 7], different TRP channels [8, 9, 10, 11,12, 13], ORAI channels [14, 15, 16], and K^+^ channels [17, 18] are required for distinct neutrophil functions. Accordingly, their inhibition or genetic ablation impairs among others neutrophil chemotaxis, recruitment, or respiratory burst.

Ion channel signaling will inevitably lead to dynamic changes of the cell membrane potential of neutrophils, which in turn feeds back on ion channels themselves by altering the electrical driving force for ion flow. Thus, the stimulation of neutrophils, for example, by chemoattractants or bacteria, is accompanied by changes in the cell membrane potential [19, 20, 21]. The cell membrane potential is a complex parameter that temporally integrates the activity of multiple ion channel types into one unified value. This principle is well exemplified by the temporal summation of inhibitory or excitatory postsynaptic potentials in neurons. By analogy with neurons, neutrophils also experience simultaneous sensory inputs that have to be combined.

The knowledge about membrane potential dynamics and its role in neutrophil function is at best only rudimentary. Several groups showed by means of flow cytometry using (voltage‐sensitive) fluorescent dyes that the membrane potential of resting human neutrophils amounts to ~−60 mV (range: −26.7 mV to −100 mV; reviewed in [6]). We recently reported a resting membrane potential of ~−75 mV in murine neutrophils using live‐cell imaging techniques [19]. This value indicates that K^+^ channels have an important share in setting the membrane potential of resting neutrophils. On the other hand, store‐operated Ca^2+^ entry following neutrophil stimulation [15] or the respiratory burst of neutrophils are accompanied by an enormous depolarization of the membrane potential. The respiratory burst may depolarize neutrophils to +60 mV [22]. Thus, the cell membrane potential of neutrophils may vary by ≥ 100 mV, which roughly corresponds to the amplitude of an action potential in excitable cells. Notably, the depolarizing currents carried by Ca^2+^ ions (e.g., via ORAI channels [15]) may elicit a faster and more transient depolarization than those by electrons during the respiratory burst triggered by PMA [19]. Changes in membrane potential will inevitably impact on multiple downstream electrogenic effectors, such as Ca^2+^ signaling [23]. Ca^2+^ influx via TRP channels or ORAI channels can only be maintained in the presence of an adequate electrochemical driving force [15]. However, so far, the functional implications of the enormous membrane potential dynamics in neutrophils as well as its underlying mechanisms are largely unknown.

Here, we focus on the role of three K^+^ channels in membrane potential dynamics and migration/chemotaxis of C5a‐stimulated neutrophils. K_Ca_3.1 [17], K_V_1.3 [18], and K_ir_2.1 [24] were reported to be expressed in neutrophils. Yet, there are only few data on the role of these K^+^ channels in membrane potential dynamics of neutrophils and their response to physiological chemoattractants, although K_V_1.3 and K_Ca_3.1 channels were reported to be required for neutrophil recruitment [18] and chemotaxis [17], respectively. K_Ca_3.1 channels were shown to hyperpolarize the membrane potential of neutrophils during artificially induced store‐operated Ca^2+^ entry [15]. K_ir_2.1 channels were identified in patch‐clamp experiments, but their function in neutrophils remains elusive [24].

Interestingly, the above‐mentioned three K^+^ channels, K_Ca_3.1, K_V_1.3, and K_ir_2.1, are also expressed in a number of other immune cells. They have emerged as important regulators of immune cell function and neuroinflammatory processes. K_Ca_3.1, in particular, is important for cell migration. Its inhibition reduces the motility of microglia [25, 26], T cells [27], and dendritic cells [28]. Moreover, K_Ca_3.1 activity was linked to T cell [29] and microglial [25] activation. The therapeutic potential of inhibiting K_Ca_3.1 channels in microglia with senicapoc is currently being tested in Alzheimer's patients [30] (clinicaltrials.gov: NCT04804241).

K_V_1.3 is likewise of immunological importance. Its upregulation is associated with autoimmune responses, while its downregulation was linked to immunodeficiency [31]. K_V_1.3 is further involved in the formation and function of memory T cells, particularly through its regulation of Ca^2+^ signaling, cytokine production, and cellular proliferation [32]. Similar to K_Ca_3.1, inhibition of K_V_1.3 in microglia was also associated with beneficial effects in Alzheimer's disease [33]. Recently, it was shown that the inhibition of mitochondrial K_V_1.3 elicits beneficial effects in a model of multiple sclerosis by selectively targeting autoreactive/autoproliferative effector memory T cells [34].

Finally, K_ir_2.1 was linked to the phenotypic differentiation of microglia [35], and it regulates macrophage migration [36]. Notably, K_ir_2.1 channels have a marked impact on the membrane potential of macrophages, which in turn modulates store‐operated Ca^2+^ entry [36] and nutrient uptake [37]. Inhibition of K_ir_2.1 channels thereby leads to impaired migration of macrophages.

Based on these considerations, the present work aims to systematically investigate the relationship between K_Ca_3.1, K_V_1.3 and K_ir_2.1 channel activity, membrane potential, and migration of C5a‐stimulated neutrophils. We focused on these channels because they stand out as important modulators of immune cell function so that their inhibition could constitute a multi‐level anti‐inflammatory approach. Here we show that K_ir_2.1 channels have the strongest impact on membrane potential dynamics of C5a‐stimulated neutrophils. This is paralleled by a marked reduction of LTB_4_ secretion and chemotaxis.

## 2 Results

### 2.1 K^+^ Channels in Membrane Potential Dynamics of Neutrophils

Membrane potential measurements were performed as described earlier using the fluorescent voltage‐sensitive dye DiBAC_4_(3) [19]. However, the time span of interest was extended from 5 to 10 min following stimulation with C5a. The aim was to evaluate the contribution of K^+^ channels to membrane potential dynamics and cell motility. We focused on three types of K^+^ channels (K_Ca_3.1, K_V_1.3, and K_ir_2.1) that are known to be widely expressed in immune cells including neutrophils [17, 18, 24].

Figure 1A depicts the time course of the membrane potential and migration velocity before and after stimulation with C5a (6 nmol/L). The resting membrane potential of untreated neutrophils was −73.1 ± 2.4 mV (Figure 1B). C5a induced a rapid, but transient depolarization up to 6.8 ± 4.0 mV within 1 min (Figure 1A,B). After 4 min, the membrane potential hyperpolarized to values more negative than −50 mV, and baseline values (−73.2 ± 1.7 mV; Figure 1B) were reached after 8 min. The transient depolarization of the membrane potential was accompanied by a transient reduction of the migration velocity. Velocity dropped from 2.6 ± 0.3 µm/min to 1.3 ± 0.1 µm/min (Figure 1B) within the first minute of C5a stimulation. Migration velocity returned to values above baseline within the next minutes (3.6 ± 0.3 µm/min; at *t* = 8 to 10 min; Figure 1B). Thus, the reduction of velocity upon C5a‐stimulation was reversible. The results suggest an inverse relationship of membrane potential and cell velocity.

**Figure 1:**
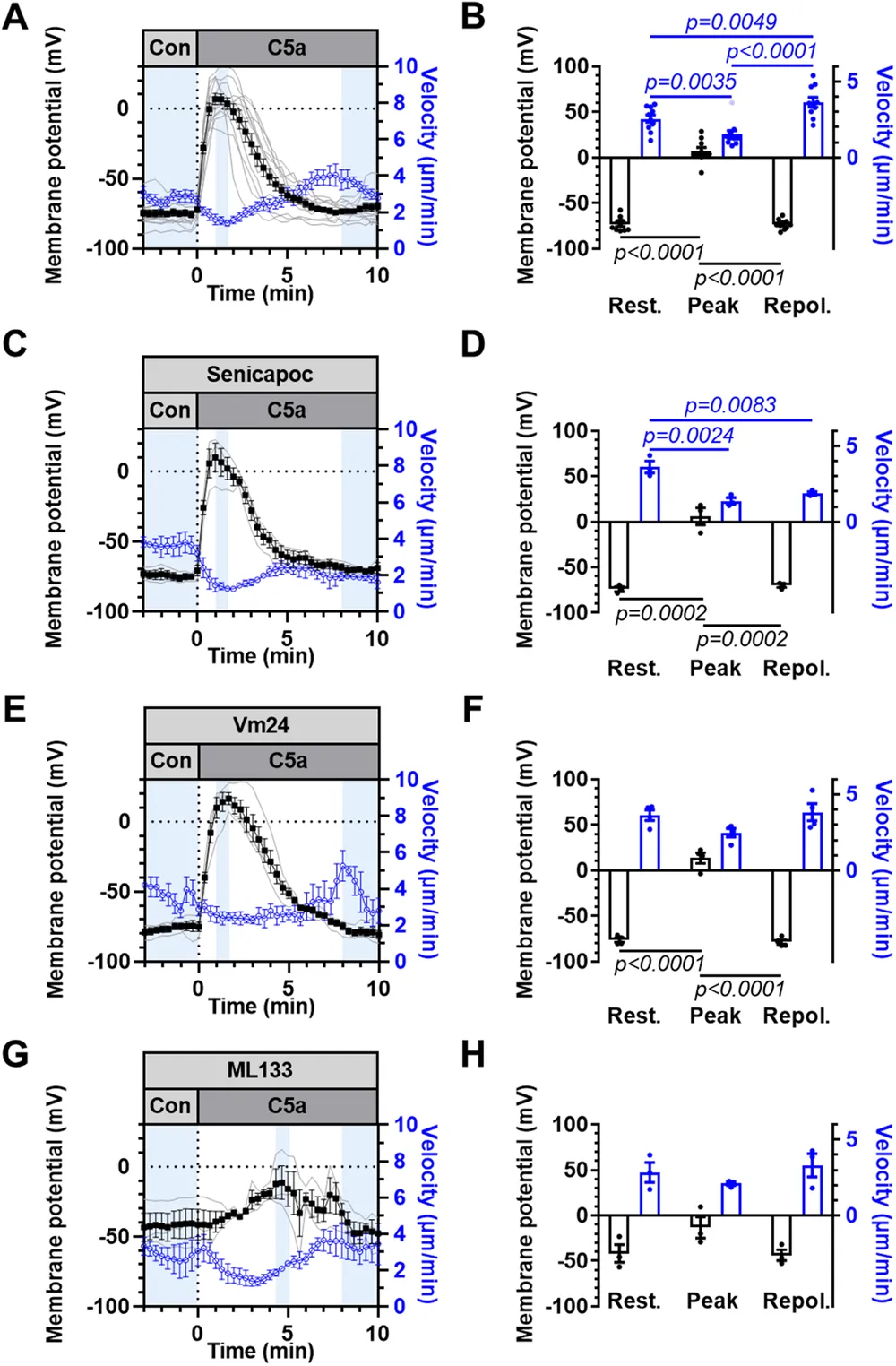
Membrane potential and migration velocity of C5a‐stimulated neutrophils in the absence and presence of different K^+^ channel inhibitors. Neutrophils were always stimulated with 6 nmol/L C5a at *t* = 0 min. K^+^ channel inhibitors were present throughout the entire experiment as indicated. The thin grey lines in panels A, C, E and G represent the mean values of neutrophils isolated from individual mice. The thick black lines illustrate the mean values derived from all individual mice. The mean values plotted in panels B, D, F and H were obtained from the time periods labeled by the shaded areas in panels A, C, E, and G. Rest., Peak and Repol. correspond to *t* = −3–0 min, maximal depolarization and *t *= 8–10 min, respectively. (A,B) Control experiments in the absence of K^+^ channel inhibitors (*N* = 12 mice; *n* = 135 cells; *n* ≥ 5 cells each). (C, D) Inhibition of K_Ca_3.1 channels with 10 µmol/L senicapoc (*N* = 4 mice; *n* = 40 cells; *n* ≥ 9 cells each). (E, F) Inhibition of K_V_1.3 channels with Vm24 (*N* = 4 mice; *n* = 42 cells; *n* ≥ 10 cells each). (G, H) Inhibition of K_ir_2.1 channels with 10 µmol/L ML133 (*N* = 3 mice; *n* = 34 cells; *n* ≥ 10 cells each). Normally distributed data were analyzed with one‐way ANOVA followed by Tukey's multiple comparison.

Figure 1C,D summarize analogous experiments in the continued presence of the K_Ca_3.1 channel blocker senicapoc (10 µmol/L). The membrane potential dynamics of senicapoc‐treated neutrophils were similar to those observed in the untreated control group. In the presence of senicapoc, the resting membrane potential was −74.0 ± 2.6 mV (Figure 1D). C5a induced a depolarization to 6.1 ± 9.3 mV within 1 min (Figure 1C,D). The membrane potential repolarized to −50 mV within 4 min, and baseline values (−70.0 ± 2.0 mV) were reached after 8 min (Figure 1D). During the resting phase, cell velocity was 3.6 ± 0.4 µm/min (Figure 1D). In the presence of senicapoc, C5a stimulation resulted in a decrease of velocity to 1.4 ± 0.2 µm/min (Figure 1D). In contrast to control conditions, velocity did not recover during the repolarisation phase, but remained at 1.9 ± 0.1 µm/min (Figure 1D). Figure 1E,F summarize experiments in the continued presence of the K_V_1.3 channel blocker Vm24 (10 nmol/L). The resting membrane potential of neutrophils superfused with Vm24 was −76.3 ± 2.3 mV. Vm24 had no impact on membrane potential dynamics of C5a‐stimulated neutrophils. C5a induced the same rapid and transient depolarization to positive values (13.4 ± 5.8 mV; Figure 1E) when K_V_1.3 channels were blocked. Membrane potential repolarized to −78.6 ± 2.5 mV at *t* = 8–10 min (Figure 1F). It is notable that there was only a trend toward reduced velocity during depolarization. Unlike senicapoc‐treated neutrophils, those treated with Vm24 reached their initial velocity upon repolarization (Figure 1F). Vm24 (10 nmo/L) could also partially inhibit K_Ca_3.1 channels [38]. However, the response to the K_V_1.3 inhibitor Vm24 is distinct from that to the K_Ca_3.1 inhibitor senicapoc. Vm24 causes no sustained decrease of the migration velocity. Therefore, we believe that the potential off‐target effect of Vm24 does not play a major role.

In the next set of experiments, we explored the role of K_ir_2.1 channels using the inhibitor ML133 (10 µmol/L; Figure 1G,H). Inhibiting K_ir_2.1 channels had a marked impact on membrane potential dynamics. The resting membrane potential was depolarized by approximately 30 mV to −41.9 ± 9.8 mV (Figure 1H). Furthermore, the C5a‐induced depolarization was attenuated. Membrane potential depolarized by less than 30 mV and reached peak values of −13.2 ± 11.7 mV (Figure 1H). Moreover, the C5a‐induced depolarization was delayed by 3 min when compared with control neutrophils (Figure 1G vs. Figure 1A). Nonetheless, the delayed depolarization was transient and returned to its resting value of −43.7 ± 6.1 mV at *t *= 8–10 min (Figure 1H). In the presence of ML133, migration velocity transiently decreased before membrane potential depolarization (Figure 1H). The velocity was 2.8 ± 0.7 µm/min, 2.1 ± 0.1 µm/min, and 3.3 ± 0.8 µm/min under control conditions, during depolarization and after repolarization, respectively (Figure 1H).

### 2.2 K_Ca_3.1 Channel Proteins are Expressed in

To reveal expression of K_Ca_3.1 channel protein in neutrophils we performed Western blot and immunostainings. These experiments revealed the presence of K_Ca_3.1 in murine neutrophils (Figure 2). We detected a band of the expected size of 47 kDa (molecular weight of K_Ca_3.1). For reference, we blotted a lysate of K_Ca_3.1‐overexpressing HEK293 cells. Immunofluorescence showed the typical punctate staining pattern of K_Ca_3.1 channels [39].

**Figure 2:**
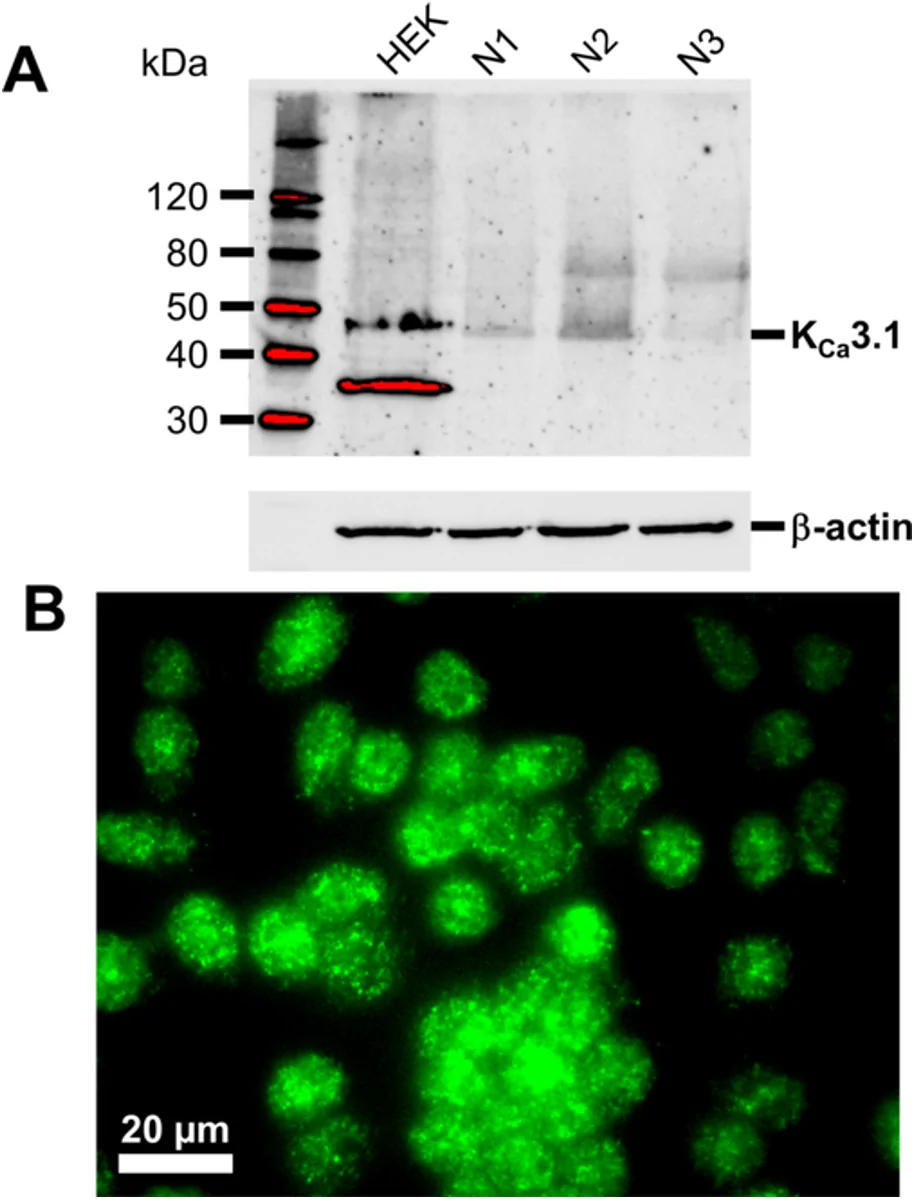
(A) Western blot detection of K_Ca_3.1 channels in murine neutrophils. Three entire neutrophil lysates from two mice each (N1–N3) were compared with a lysate from K_Ca_3.1‐overexpressing HEK293 cells (HEK). The β‐actin blot served as a loading control. (B) The same K_Ca_3.1 antibody was used for immunostaining of murine neutrophils and showed the typical punctate staining pattern of K_Ca_3.1 channels. Scale bar: 20 µm.

### 2.3 K^+^ Channel Inhibition Impairs Neutrophil Chemotaxis

Here we analyzed the impact of the three K^+^ channels on neutrophil motility in a more physiological setting by placing neutrophils in a three‐dimensional collagen gel and exposing them to a chemotactic gradient of C5a. We determined the migration velocity and the chemotaxis index. The results of these experiments are shown in Figure 3.

**Figure 3:**
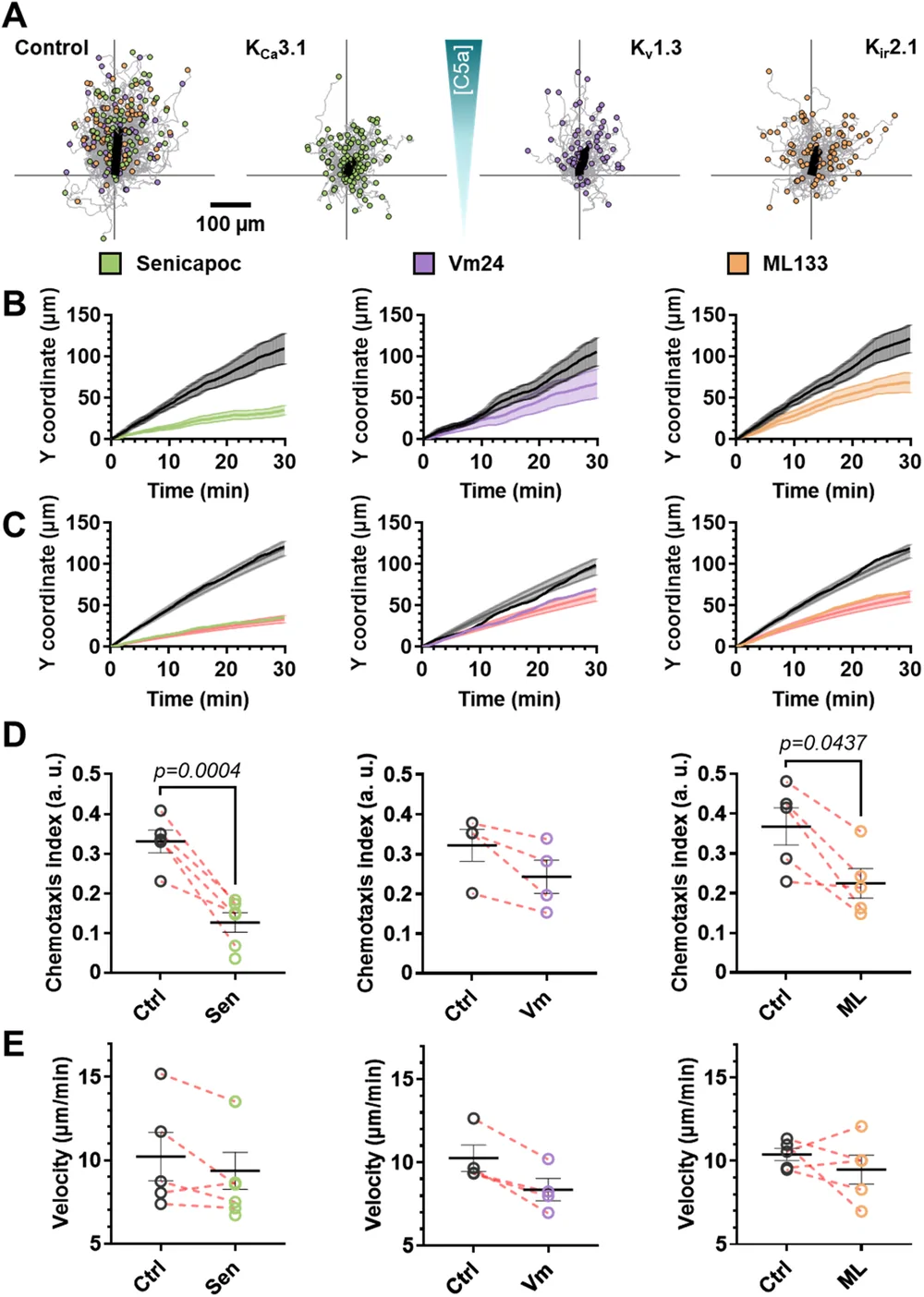
Chemotaxis of neutrophils in the presence of different K^+^ channel inhibitors. (A) Trajectories of chemotacting neutrophils. The trajectories of individual neutrophils are represented by thin grey lines with their endpoints being marked by a coloured dot. The thick lines correspond to the mean trajectories of the respective cell populations. Controls are grouped in a single chart (left panel). Scale bar: 100 µm. (B–E) Summary of the chemotaxis experiments. (B) Movement into the direction of the C5a gradient (“Y coordinate”). The dark and colored curves illustrate the movement of neutrophils in the absence and presence of K^+^ channel blockers, respectively. (C) Data derived from the simulations performed using the parameters in Table 1 (±SD). For comparison, we also plotted the mean values of the original data using the same colors as shown in B. (D) Mean chemotaxis indices. Each dot reflects the mean value of neutrophils isolated from one mouse. The dotted lines connect data derived from one mouse. (E) Mean migration velocity. Each dot reflects the mean value of neutrophils isolated from one mouse. The dotted lines connect data derived from the same mouse. Statistical analysis was performed by an unpaired t‐test for normally distributed data, or Mann‐Whitney if otherwise (*N* = 4–6; *n* = 61–125).

Figure 3A displays the trajectories of individual neutrophils in a C5a gradient in the absence and presence of the K^+^ channel inhibitors. The thick black line corresponds to the mean path of the respective neutrophil populations. The length of the bold black line reflects chemotactic efficiency, which is reduced in the presence of the K^+^ channel blockers. Figure 3B–E provide a detailed summary of these experiments. Blocking K_Ca_3.1 with senicapoc produced marked effects on neutrophil chemotaxis. Migration into the direction of higher C5a concentrations (“Y coordinate”) decreased from 109.5 ± 18.4 µm (*N/n* = 5/75) to 35.1 ± 5.7 µm (Figure 3B, left panel; *N/n* = 6/125). Accordingly, the chemotaxis index was reduced from 0.33 ± 0.03 to 0.13 ± 0.02 (Figure 3D, left panel). In contrast, velocity of migration remained largely unaffected, with values of 10.2 ± 1.4 µm/min under control conditions and 9.4 ± 1.1 µm/min in the presence of senicapoc (Figure 3E, left panel). Thus, K_Ca_3.1 inhibition affected primarily chemotaxis.

Inhibiting the K_V_1.3 channel with Vm24 also reduced directed migration. In the presence of Vm24 neutrophils covered only 67.5 ± 17.7 µm (*N/n* = 4/65) into the direction of the C5a gradient as opposed to 105.6 ± 17.1 µm under control conditions (Figure 3B, middle panel; *N/n* = 4/61). The chemotaxis index fell from 0.32 ± 0.04 to 0.24 ± 0.04 (Figure 3D, middle panel). Mean velocity was reduced across all experiments, declining from 10.2 ± 0.8 µm/min to 8.4 ± 0.7 µm/min (Figure 3E, middle panel).

Inhibition of K_ir_2.1 channels resulted in a reduction in migration along the C5a gradient (Y coordinate) over time by approximately 50% (Figure 3B, right panel). Consistently, the chemotaxis index decreased from 0.37 ± 0.05 (*N/n* = 5/85) to 0.22 ± 0.04 (Figure 3D; right panel; *N/n *= 5/65). Notably, the movement into the direction of the C5a gradient was particularly low toward the end of the experiment. In the last 5 min of the experiment neutrophils advanced only ~2 µm along the C5a gradient in the presence of ML133 as opposed to ~11 µm under control conditions. Such a progressive nature of the decrease of chemotaxis was not seen when K_Ca_3.1 or K_V_1.3 channels were inhibited. In contrast, velocity remained essentially unchanged, with 10.4 ± 0.4 µm/min under control conditions and 9.5 ± 0.9 µm/min in the presence of the K_ir_2.1 blocker ML133 (10 µmol/L; Figure 3E, right panel).

To obtain a more detailed and subtle view on the role of K^+^ channels in neutrophil migration and chemotaxis, we applied a mathematical model analysis recently developed [40]. It is based on a stochastic model of fractional Brownian motion with exponential tempering. The key parameters derived from this analysis are summarized in Table 1. The validity of the model‐based analysis was shown by simulating the movement along the chemotactic gradient (Y coordinate) that overlapped with the original data (see Figure 3C). The data reveal that K_Ca_3.1, K_V_1.3 and K_ir_2.1 channels had a differential impact on neutrophil chemotaxis toward C5a. Chemotaxis was less anomalous when K_Ca_3.1 and K_ir_2.1 channels were blocked; the diffusion coefficient was reduced to a similar extent by all blockers, and the drift velocity depended most strongly on K_Ca_3.1 channels. Overall, K_V_1.3 channels appeared to have the smallest effect on neutrophil chemotaxis toward C5a.

**Table 1:** Summary of key parameters obtained from the mathematical model analysis. The para meters are calculated for the movement into the direction of the C5a gradient (Y coordinate).

	*H*	*D* (µm^2^/min^2H^)	*v*_*d*_ (µm/min)
Ctrl‐Sen	0.742 ± 0.007	28.4 ± 1.2	4.9 ± 0.5
Sen	0.696 ± 0.005	15.3 ± 0.4	1.5 ± 0.3
Ctrl‐Vm24	0.843 ± 0.006	35.0 ± 1.8	3.7 ± 0.7
Vm24	0.829 ± 0.006	22.9 ± 1.1	2.5 ± 0.3
Ctrl‐ML133	0.858 ± 0.006	38.8 ± 1.8	4.8 ± 0.5
ML133	0.812 ± 0.005	27.8 ± 1.1	3.0 ± 0.5

Abbreviations: Ctrl‐, control experiments performed with neutrophils from the same mice as those with inhibitors; *D*, diffusion coefficient, a measure of the spreading behavior of neutrophils; *H,* Hurst coefficient, a measure of anomality; v_d_, drift velocity of neutrophils into the direction of the chemotactic gradient; ML133, K_ir_2.1 inhibitor; Sen, K_Ca_3.1 inhibitor senicapoc; Vm24, K_V_1.3 inhibitor.

### 2.4 Leukotriene B_4_ Quantification

Neutrophil chemotaxis toward C5a depends on the amplification by the secretion of LTB_4_ [2, 3]. We therefore tested whether impaired chemotaxis in the presence of K^+^ channel blockers could be explained by reduced LTB_4_ secretion. Figure 4 provides a summary of these experiments. Our experiments corroborated prior findings. LTB_4_ secretion rose in response to C5a‐stimulation by a factor of 2.5 (resting: 1172 ± 156 pg/mL (*N* = 4) *vs.* C5a‐stimulation: 2954 ± 461 pg/mL (*N* = 4)). Subsequently, we analyzed the impact of K^+^ channel inhibition on C5a‐induced LTB_4_ secretion in neutrophils. K_Ca_3.1 inhibition reduced LTB_4_ secretion by almost 50% (1623 ± 261 pg/mL; *N* = 4), while K_V_1.3 inhibition with Vm24 had no effect on LTB_4_ secretion (2511 ± 403 pg/mL; *N* = 4). Inhibition of K_ir_2.1 channels with ML133 resulted in the strongest reduction of the LTB_4_ concentration in the supernatants to levels comparable to those observed in unstimulated cells (824 ± 392 pg/mL; *N* = 4). Notably, the combined application of all three K^+^ channel inhibitors led to an LTB_4_ concentration approximating the baseline level or that obtained by the addition of ML133 alone (948 ± 48 pg/mL; *N* = 4).

**Figure 4:**
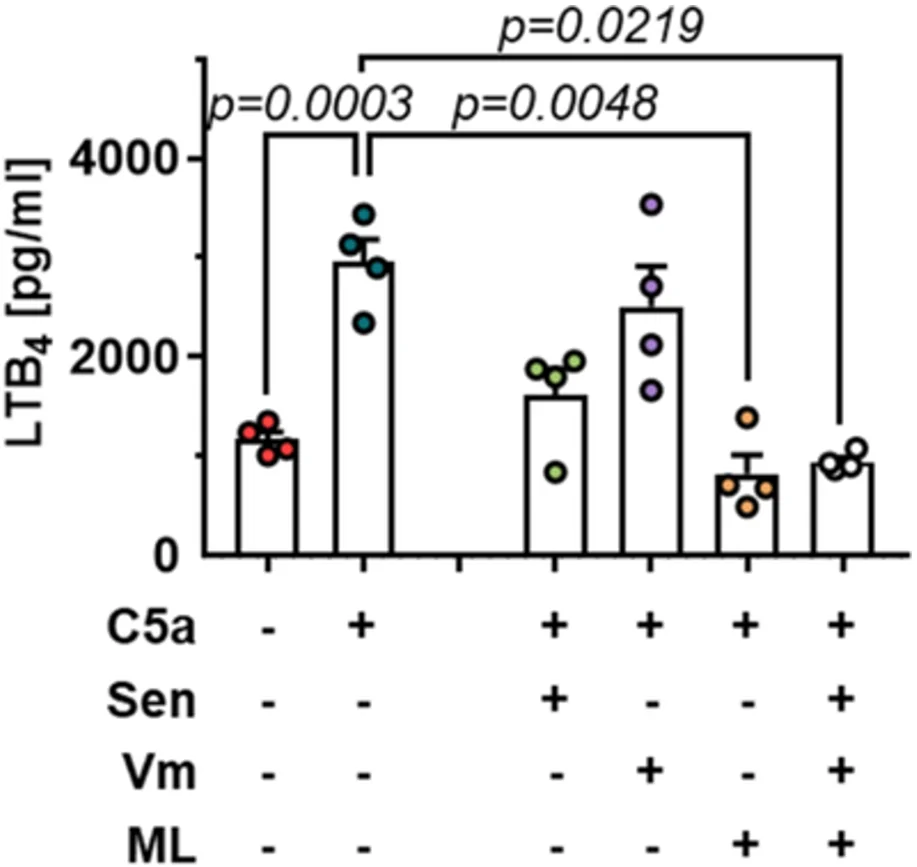
Leukotriene B_4_ (LTB_4_) concentrations in supernatants of murine neutrophils. Cells were exposed to C5a in the absence and presence of K^+^ channel inhibitors as indicated for 1 h. Statistical analysis was performed using the Kruskal–Wallis test (*N* = 4).

## 3 Discussion

Our study investigated the interplay of K^+^ channels, membrane potential dynamics, and migration of neutrophil granulocytes. Upon C5a stimulation, neutrophils undergo a rapid and transient depolarization by ~90 mV, which coincides with a marked reduction in migration velocity. The subsequent recovery of motility upon hyperpolarization suggests a functional coupling between membrane potential and migratory capacity. Remarkably, such a behavior could also be observed during the calibration of the membrane potential measurements. We always started the calibration by clamping the membrane potential to ~−80 mV. This step was frequently accompanied by a sudden increase of the migration velocity. Thus, our data support the idea that dynamic regulation of the membrane potential is not merely a passive consequence of cellular activation but an active determinant of neutrophil behavior. The recovery of the migration velocity following hyperpolarization implies that restoration of a negative membrane potential is necessary for re‐establishing optimal intracellular signaling conditions. Yet, a negative membrane potential alone appears not to be sufficient. In particular, K_Ca_3.1 channels have to be in an un‐inhibited state because K_Ca_3.1 blockade with senicapoc precluded the recovery of the migration velocity upon repolarization, although the membrane potential did not differ from control conditions.

The inhibition of either K_Ca_3.1, K_V_1.3, or K_ir_2.1 channels caused distinct and non‐redundant effects. K_ir_2.1 clearly emerged as a key regulator of the resting membrane potential of neutrophils. Its inhibition resulted in a marked and sustained depolarization of the resting membrane potential, delayed depolarization and impaired repolarization in response to C5a. A similar role of K_ir_2.1 channels in maintaining the resting membrane potential was found in other immune cells such as macrophages and microglia [36, 41]. Notably, while migration velocity remained largely unaffected in the presence of the K_ir_2.1 inhibitor ML133, chemotaxis was increasingly inhibited. The dissociation between velocity and chemotaxis suggested that directional sensing, rather than motility, is particularly sensitive to disruptions in membrane potential homeostasis. One plausible explanation is a reduction in LTB_4_ secretion, which is known to amplify paracrine chemotactic signaling [2, 3]. Depolarization of the resting membrane potential upon K_ir_2.1 inhibition reduces the driving force for Ca^2+^ influx [36, 41]. The production of LTB_4_, in turn, depends in part on Ca^2+^‐sensitive enzymes [42]. Hence, reducing the intracellular Ca^2+^ concentration by depolarizing the membrane potential is anticipated to limit LTB_4_ synthesis in neutrophils. Consistent with the dominant role of K_ir_2.1 channels, the combined inhibition of all three K^+^ channels did not further decrease LTB_4_ levels beyond those observed with unstimulated neutrophils or ML133 alone.

We are aware that the impact of ML133 on neutrophil behavior has to be discussed at the background of its specificity for K_ir_2.1 channels. To the best of our knowledge, K_ir_2.1 is the only K_ir_ channel shown to be functionally expressed in murine neutrophils. Moreover, K_ir_2.1 mRNA expression in murine neutrophils is by far the highest of all K_ir_2.x family members (e.g., Heng et al., [43] https://rstats.immgen.org/Skyline_microarray/skyline.html), also when compared with other immune cells. Thus, even if other K_ir_2.x channels were expressed in neutrophils, their contribution would be expected to be rather low. Notably, Masia et al. did not detect any mRNAs of K_ir_2.2‐2.4 in murine neutrophils by means of PCR. K_ir_7.1 and K_ir_4.1 mRNA appear to be present in murine neutrophils, albeit at a much lower level than that of K_ir_2.1. Moreover, IC_50_ values of ML133 for inhibiting these channels are much higher than 10 µM [44]. Therefore, we are confident that K_ir_2.1 was the predominant effector of ML133 in murine neutrophils in our experiments.

The K_Ca_3.1 inhibitor senicapoc was used in a seemingly very high concentration (10 µmol/L). However, we would like to point out that senicapoc was employed in a “protein‐rich” environment, in particular when studying chemotaxis. For these experiments, neutrophils were embedded in a 3D collagen matrix. This is relevant because 98% of senicapoc or related compounds are protein‐bound (B. Wünsch and C. Kick: personal communication; [45]). Thus, the free senicapoc concentration was ~500 nM in our experiments, which would cause a ~95% inhibition of K_Ca_3.1 channels [46]. K_Ca_3.1 channels did not contribute to the maintenance of the resting membrane potential and appeared to be inactive under basal conditions, which is in line with their Ca^2+^‐dependent gating properties. Furthermore, they did not affect C5a‐induced membrane potential dynamics. Yet, the impact of K_Ca_3.1 inhibition became apparent at the level of migration: Inhibition of K_Ca_3.1 prevented the recovery of migration speed following depolarization, reduced chemotaxis, and tended to decrease LTB_4_ secretion. Thus, K_Ca_3.1 channels seemed to regulate migration and chemotaxis of neutrophils in a way that appeared to be independent of the membrane potential. Non‐conductive properties or an altered regulation of cell volume could come into play. Cell volume is a known determinant of (neutrophil) migration [47, 48, 49, 50]. K_Ca_3.1 channels are also expressed in the inner membrane of mitochondria. In lung cancer cells the inhibition of mitoK_Ca_3.1 channels decreased mitochondrial ATP production and inhibited cell motility [51]. Mitochondrial ATP, in turn, was shown to regulate neutrophil chemotaxis [52]. To assess the function of mitoK_Ca_3.1 channels more directly, we developed a mitochondria‐targeted senicapoc derivative. While this compound was highly effective in lung and breast cancer cells, it elicited hardly any effect on neutrophil chemotaxis (unpublished observations from our laboratory). Therefore, it is unlikely that senicapoc, which also inhibits mitochondrial K_Ca_3.1 channels, impaired neutrophil chemotaxis via this mechanism.

K_V_1.3 channels played no role in membrane potential regulation of C5a‐stimulated neutrophils either. Based on the biophysical properties of K_V_1.3 channels, in particular their rapid inactivation within less than a second [53], this observation was not entirely unexpected. Thus, although K_V_1.3 channels could in principle be activated by the C5a‐induced depolarization, they would be immediately inactivated so that their impact on the cell membrane potential is strongly mitigated. Nevertheless, our experiments revealed a small impact on chemotaxis and migration. This effect is unlikely to be mediated by direct electrical regulation, but might instead reflect non‐conductive functions. Previous studies demonstrated interactions between K_V_1.3 channels and *β*1‐integrins [54, 55]. Given that *β*1‐integrins interact with collagen I, as used in our chemotaxis assays, such interactions could provide a possible explanation for our observations. Moreover, recent findings have found a role for K_V_1.3 channel proteins as a cellular signaling hub [56]. Notably, K_V_1.3 inhibition did not affect LTB_4_ production, and Vm24 does not reach mitochondrial K_V_1.3 channels. Vm24 could have caused a partial inhibition of K_Ca_3.1 channels when used at a concentration of 10 nM [38]. However, if such off‐target effects were responsible for the minor impact of Vm24 on neutrophil behavior, it would confirm our conclusion that K_V_1.3 channels play only a small role in neutrophil chemotaxis. In any case, K_V_1.3 channels impacted neutrophil motility in a way that was distinct from that of K_ir_2.1 or K_Ca_3.1 channels.

## 4 Conclusion

Our study identified membrane potential dynamics as an important regulatory axis in neutrophil migration and disclosed distinct contributions of specific K^+^ channels. By linking electrical signaling to chemotactic amplification via LTB_4_, our findings provide a concept for understanding how K_ir_2.1 channel activity orchestrates neutrophil behavior. Moreover, our findings provide evidence that K_Ca_3.1 and K_V_1.3 channels may affect neutrophil behavior by their non‐conductive properties.

## 5 Methods

### 5.1 Cell Culture

WEHI‐3B cells were cultured in DMEM supplemented with 10% fetal calf serum, 100 U/mL penicillin/100 µg/mL streptomycin and glutamine at 37°C and 5% CO_2_ until they reached 80% confluence. The supernatant was collected, sterile‐filtered to remove residual cells, and stored in aliquots at −20°C for later use. For passaging, the cells were washed with prewarmed PBS (37°C), detached by incubation with 1 mL Trypsin‐EDTA (37°C), and the reaction was stopped with 10 mL fresh WEHI medium. The WEHI‐3B cell suspension was centrifuged at 200 g for 10 min. Afterwards, the cells were resuspended and subcultured.

### 5.2 Isolation of Neutrophils From Mice

The use of mice was reported to the local authorities (T25.003.UMS). Bone marrow neutrophils were isolated from C57BL/6 mice (both sexes, 8–24 weeks old). Animals were euthanized by cervical dislocation. Bone marrow of femurae and tibiae was centrifuged into wash buffer (HBSS^−/−^, 20 mM HEPES, 5% FCS). Cell suspensions were filtered through a 70 µm strainer and centrifuged at 200 g for 10 min. Pellets were resuspended in HBSS^−/−^ (20 mM HEPES) and layered onto a discontinuous Histopaque 1077/1119 gradient (PAN Biotech, Germany). After centrifugation (650 g, 30 min, no brake), neutrophils were collected from the interphase, washed twice, and resuspended in RPMI 1640 supplemented with 10% FCS, 10% WEHI‐3B‐conditioned medium, 100 U/mL penicillin and 100 µg/mL streptomycin. Neutrophils were incubated overnight at 37°C and 5% CO_2_.

### 5.3 Simultaneous Measurement of Membrane Potential and Cell Migration

Membrane potential and cell motility were measured as described previously [19]. After overnight incubation, neutrophils were pelleted (200 g, 10 min, 25°C) and resuspended in 1 mL pre‐warmed (37°C) Ringer's solution (122.5 mM NaCl, 1.2 mM CaCl_2_, 0.8 mM MgCl_2_, 5.4 mM KCl, 10 mM HEPES, 5.5 mM glucose, pH was adjusted with 1 N NaOH), that was complemented with 2 µM DiBAC_4_(3) (AAT Bioquest, USA). Suspended neutrophils were plated on fibronectin‐coated (25 µg/mL) ibidi µ‐slides (type I) and incubated in a warming cabinet at 37°C for 10 min.

Thereafter, ibidi µ‐slides were placed on the heated stage (37°C) of an inverted fluorescence microscope (Zeiss Axiovert 200, 40× oil objective) equipped with a pco.edge sCMOS camera. Neutrophils were continuously superfused with DiBAC_4_(3)‐containing Ringer's solution. DiBAC_4_(3) fluorescence was excited at 490 nm every other 20 s using a VisiChrome High‐Speed Polychromator (Visitron Systems, Germany). Emission was measured at 520 nm. Data were acquired with VisiView software.

Measurements began with a 3 min control period during which cells were superfused with Ringer's solution. When indicated, we added either senicapoc (K_Ca_3.1 inhibitor; 10 µM, kindly provided by Christina Kick, Institut für Pharmazeutische und Medizinische Chemie, University Münster), Vm24 (K_V_1.3 inhibitor; 10 nM [57]) or ML133 (K_ir_2.1 inhibitor; 10 µM, Tocris Bioscience, UK) to the Ringer's solution. Subsequently, neutrophils were stimulated with C5a (6 nM; Biotechne, USA) for 10 min in the absence or presence of one of the K^+^ channel blockers. Thereafter, neutrophils were sequentially superfused with the following modified Ringer's solutions for calibration: Ringer's solutions contained 2 mM, 35 mM, and 140 mM NaCl. NaCl was isosmotically replaced with NMDG‐Cl when required. Calibration solutions contained 2 µg/mL gramicidin. All solutions employed during the experiment were supplemented with DiBAC_4_(3) (2 µM).

Background‐corrected fluorescence intensity of individual neutrophils was measured by segmenting their total cell area with ImageJ. Absolute membrane potential values were calculated using the Goldman–Hodgkin–Katz equation. Intracellular Na^+^ concentration is 12 mM Na^+^ [11], and we assumed an intracellular K^+^ concentration of 140 mM. Fluorescence intensities for each neutrophil were transferred to membrane potential by linear regression (*r* ^2^ ≥ 0.9). To compare different conditions, three time intervals were defined: Resting phase (Rest.), from *t* = −3 to 0 min; peak phase, defined as the time point at which the mean membrane potential reached maximal depolarization plus 1 min; repolarisation phase (Repol.), from t = 8 to 10 min.

Cell migration was quantified by calculating the *x–y*‐coordinates of the centroids throughout the entire image stack acquired during membrane potential measurements. Thereby, membrane potential and cell motility could be determined simultaneously. Velocity was determined as a three‐point difference quotient [58].

### 5.4 Preparation of Vm24

Recombinant Vm24 was produced as previously described [57]. Briefly, a *Pichia pastoris* strain transformed for Vm24 expression was cultured overnight in YPD medium (1% yeast extract, 2% peptone, and 2% dextrose; pH 7.0) supplemented with 200 µg/mL Zeocin. The culture was subsequently diluted in BMGY medium (1% yeast extract, 2% peptone, 100 mM potassium phosphate, pH 6.0, 1.34% yeast nitrogen base [YNB], 4 × 10^−5^% biotin, and 2% glycerol) and incubated at 30°C with shaking at 240 rpm for 24 h. Cells were harvested by centrifugation, resuspended in BMMY induction medium (identical to BMGY except that glycerol was replaced with 1% methanol), and cultured for 72 h at 30°C with continuous shaking at 240 rpm. To maintain protein induction, absolute methanol was added every 24 h to a final concentration of 1%.

Recombinant Vm24 was purified using a two‐step protocol. Briefly, the culture supernatant was filtered and applied to a manually packed Ni Sepharose 6 Fast Flow affinity column (Cytiva), taking advantage of the His‐tag fused to the recombinant protein. Following the washing step, Vm24 was eluted from the affinity column and further purified by reverse‐phase high‐performance liquid chromatography (RP‐HPLC) using a C18 analytical reverse‐phase column. Elution was performed with a linear gradient of 10%–40% solvent B (0.1% trifluoroacetic acid [TFA] in 100% acetonitrile) in solvent A (0.1% TFA in water) over 60 min at a flow rate of 1 mL/min.

### 5.5 Immunostaining

Glass‐bottom dishes were coated with fibronectin (25 µg/mL in RPMI) at room temperature for 30 min and washed once with PBS^(+/+)^ (Mg^2+^/Ca^2+^). After overnight incubation, 250,000 neutrophils were plated on fibronectin‐coated glass bottom dishes and incubated at 37°C for 10 min. Thereafter, neutrophils were fixed with 3.5% paraformaldehyde (PFA) at room temperature for 30 min. After two washes with PBS^(^^−/−)^, neutrophils were permeabilized with 0.25% Triton X‐100% and 1% SDS in PBS at room temperature for 10 min. Following three additional washes with PBS^(^^−/−)^ neutrophils were blocked with 10% FCS in PBS for 30 min. Neutrophils were then incubated with the primary antibody (anti‐K_Ca_3.1, Sigma; 1:300 in 10% FCS/PBS) at room temperature in a humidified chamber for 1 h. After thorough washing, the secondary antibody (goat anti‐rabbit Alexa Fluor 488 Invitrogen; 1:1000 in 10% FCS/PBS) was applied at room temperature for 45 min. Then neutrophils were washed again and fixed with 3.5% PFA for 10 min. Images were acquired using a fluorescence microscope (Axiovert 200, 100x objective; Zeiss, Oberkochen, Germany) equipped with a FITC filter set.

### 5.6 Western Blot

Neutrophils isolated from two mice were pooled and cultured overnight. The following day, neutrophils were pelleted, washed with PBS, centrifuged, and lysed in 150 µL RIPA buffer. Rotation at 4°C for 20 min. Lysates were cleared by centrifugation (14,000 G, 4°C, 15 min) and stored at −70°C. Protein concentrations were determined using the Pierce BCA Protein Assay Kit (Thermo Fisher Scientific) according to the manufacturer's instructions. Proteins from the entire lysate were separated by SDS‐PAGE using a 10% acrylamide resolving gel and transferred onto a nitrocellulose membrane Thermo Fisher Scientific) with a semi‐dry Trans‐Blot Turbo system (Bio‐Rad; 7 min; 13 A; 25 V). Membranes were blocked with 5% skim milk in TBS‐Tween (Tris‐buffered saline + Tween20) for 1 h and incubated at 4°C overnight with anti‐K_Ca_3.1 antibody (Sigma; 1:300 in 5% milk). After three washes with TBS‐Tween, blots were incubated with HRP (Horseradish peroxidase; 1:10,000) conjugated goat anti‐mouse secondary antibody at room temperature for 1 h. Following additional washes, bands were visualized using Clarity Max Western ECL substrate (Bio‐Rad) and imaged with a ChemiDoc system (Bio‐Rad).

### 5.7 3D Chemotaxis Assay

Chemotaxis in three‐dimensional collagen gels was assessed as described previously [2]. We used µ‐slides Chemotaxis (ibidi, Gräfelfing, Germany). The chemotaxis channel was first coated with fibronectin (25 µg/mL; Roche, Mannheim, Germany) at room temperature for 1 h. Following overnight incubation, neutrophils were resuspended in Ringer's solution and mixed with rat‐tail collagen I (final concentration 3.8 mg/mL; Corning, New York, USA) and HEPES‐buffered (15 mM) HBSS containing 0.79 mM Ca^2+^ and 0.57 mM Mg^2+^ (HBSS^+/+^). The cell‐collagen suspension was injected into the chemotaxis channel and allowed to polymerize at 37°C for 12 min. Then both reservoirs of the µ‐slides were filled with Ringer's solution. The Ringer's solution in one of the reservoirs was supplemented with C5a (60 nM; Bio‐techne, Minneapolis, USA). When applicable, the K^+^ channel inhibitors were added to both reservoirs as well as to the chemotaxis channel in order to achieve a homogeneous concentration. K^+^ channel blocker concentrations were: Senicapoc (10 µM), Vm24 (10 nM), and ML133 (10 µM).

Neutrophil chemotaxis was monitored at 37°C with live‐cell imaging using a phase contrast microscope (Axiovert 40 C; Zeiss, Oberkochen, Germany) equipped with a camera (Bresser MikroCam SP 3.1; Bresser, Rhede, Germany). Images were acquired in 5‐s intervals for 30 min. Image acquisition started 20 min after establishing the gradient.

Chemotaxis was quantified from the movement of the cell center over time. To this end, coordinates of the cell center were obtained in each frame of the image stack with Amira software (Thermo Fisher Scientific, Waltham, MA, USA). The following parameters were quantified: Velocity, calculated as a three‐point difference quotient; chemotaxis index (CI), defined as the quotient of net displacement parallel to the chemoattractant gradient and the total distance covered with time.

### 5.8 LTB_4_ Quantification

Leukotriene B_4_ (LTB_4_) concentrations in supernatants of neutrophils were determined using the LTB_4_ ELISA Parameter Assay Kit (Bio‐Techne, MN, USA). Neutrophils (10^6^ cells/mL) were stimulated with 60 nM C5a at 37°C for 1 h, either in the absence or presence of a single or a combination of K^+^ channel inhibitors. K^+^ channel blocker concentrations were: Senicapoc (10 µM), Vm24 (10 nM), and ML133 (10 µM). Next, the neutrophils were centrifuged, and supernatants were further processed according to the manufacturer's instructions. Standard solutions provided in the kit were used for calibration. Each biological sample was measured in two technical replicates. The absorbance was recorded at 450 nm using a microplate reader (Tecan Spark; Tecan, Männedorf, Switzerland).

### 5.9 Statistical Analysis

“N” and “n” stand for the number of mice and the number of individual neutrophils, respectively. All data are presented as mean ± standard error of the mean (SEM), unless stated otherwise. Data normality was assessed using the Shapiro–Wilk Test. For comparisons between two groups, statistical significance (*p* < 0.05) was determined using a paired or unpaired Student's *t*‐test, when data were normally distributed. Mann–Whitney *U* test was used for non‐parametric data. More than two groups with normal distribution were analyzed by one‐way analysis of variance (ANOVA) followed by a Tukey post‐hoc test and Kruskal–Wallis test followed by a Dunn's post‐hoc test for non‐parametric data. All statistical tests were performed in GraphPad Prism version 8 for Windows (GraphPad Software, Boston, MA, USA).

### 5.10 Mathematical Model Analysis

A further quantification of the dynamics of chemotacting neutrophils was performed using a mathematical model analysis based on the application of stochastic models, as described previously [40]. We applied fractional Brownian motion with exponential tempering to model the spreading of the cell groups. This model contains three parameters *H*, *D*_*H*_, and *τ* ^*^: The Hurst coefficient *H* classifies the anomality of motion (H = ½ normal diffusion, H > ½ superdiffusion, H < ½ subdiffusion). The generalized diffusion coefficient *D*_*H*_ quantifies the strength of spreading, while the time scale *τ* ^*^ defines the transition time of the mean square displacement from an anomalous (~*t* ^*2H*^) scaling to normal diffusion (~*t*) over long times. Directed movement of the cell ensembles was quantified by a time‐dependent drift $\langle y(t)\rangle =v_{d}\;{\tau \;}_{V}\left(1-e^{-t/\tau_{v}}\right)$ with two additional parameters, the drift velocity $v_{d}$ and a time scale ${\tau \;}_{V}$.

## Conflicts of Interest

The authors declare no conflicts of interest.

## Data Availability

The data that support the findings of this study are available from the corresponding author upon reasonable request.
